# Effects of constructal theory on thermal management of a power electronic system

**DOI:** 10.1038/s41598-020-78566-x

**Published:** 2020-12-08

**Authors:** Amin Asadi, Farzad Pourfattah

**Affiliations:** 1 Institute of Research and Development, Duy Tan University, Da Nang, 550000 Vietnam; 2 Faculty of Natural Sciences, Duy Tan University, Da Nang, 550000 Vietnam; 3 Department of Mechanical Engineering, Kashan University, Kashan, Iran

**Keywords:** Electrical and electronic engineering, Mechanical engineering

## Abstract

Thermal management of power electronics (PE) systems is a long-lasting challenge in their industrial applications. It is important to provide a uniform temperature distribution on the surface of the insulated gate bipolar transistors and diodes. The thermal management of a PE module is the main objective of the present study. The flow characteristics and the effects of the constructal theory on the heat transfer performance of the cooling system have been numerically investigated. The governing equations have been discretized using the finite volume method, and validation has been done to make sure the results are reliable. The effects of different channels configurations on decreasing the chips’ temperature and uniform temperature distribution on the chips’ surface have been studied. The flow characteristics and heat transfer performance at different mass flow rates for different channel configurations have been studied by presenting the results of the average chips’ temperature, Nusselt (Nu) number, pressure loss, and standard temperature deviation. Moreover, different temperature distribution contours have been presented to show the performance of different configurations. The results revealed that by changing the channels’ configuration from the conventional straight channel to leaf-inspired channels (case B and C), the cooling performance is improved.

## Introduction

It is known that thermal management is a long-lasting challenge in electronics-related industries such as power electronics (PE) systems, battery, and wearable electronics^1–5^. The applications of PE systems, which use for controlling and converting electrical energy, have been rapidly grown during the last decades. The trend of PE systems is continuing towards miniaturization and, as a result, high-performance thermal management systems are demanding, especially for insulated gate bipolar transistors (IGBTs). The most important factors affecting the technical progress and development of IGBTs are operating temperature, dimension, reliability, efficiency, and cost. With the development of IGBTs over the last thirty years, their power density has been lifted from 35 kW/cm^2^ (in the early beginning) to 250 kW/cm^2^ expected by 2020s. Because of high voltage, current, and miniaturization, the total heat dissipation of IGBTs follows an increasing trend. For example, the generated heat flux of IGBTs used in hybrid electric vehicles (HEV) is between 100 and 200 W/cm^2^ during normal operation and is expected to reach 500 W/cm^2^ in the next generation of IGBTs^6,7^.

Generating high heat fluxes by IGBTs results in facing higher and non-uniform temperature on IGBT chips, which results in considerable degradation of the device performance and system reliability. Thus it is highly demanding to have an effective cooling system to cool down the IGBTs. Moreover, it is known that an effective cooling solution results in enhancing the reliability of the device^8–10^.

One of the most important factors in thermal management of PE systems is to provide uniform temperature distribution on IGBT chips^11,12^. Although increasing the heat transfer rate leads to decreasing the IGBT’s temperature, it has no considerable effect on the temperature distribution of the IGBT surface. Thus having a cooling system that can cool down the IGBT chips below the maximum junction temperature (125 °C for silicon-based IGBT and 200 °C for silicon carbide-based IGBT) by providing a uniform temperature distribution on the IGBT chips surface is highly desirable in thermal management of PE systems. Researchers tried to tackle this problem by introducing the constructal theory. The constructal theory is the view that the generation of flow configuration is a physics phenomenon that can be based on physics principles^13^. This theory pays attention to the geometry and arrangement of the system structure and, by optimizing the geometry and structure, maximizes the performance and efficiency of the system^14^. Bejan was the first to apply structural theory in 1996^13^. One of the samples of the Constructal theory is network structures, which were first used to cool electronic components^13,15,16^. It can provide a more uniform temperature distribution compared to conventional MCHSs.

In this regard, Tan et al.^17^ conducted a numerical and experimental study on the effects of the constructal theory of different fractal-like microchannels in heat transfer performance in cooling an electronic chip with the heat flux of 100 W/cm^2^. Their results showed that the heat transfer performance of the fractal-like microchannel is by far better than the straight conventional microchannel; the fractal-like microchannel reached the maximum heat source temperature of 10 °C less than the straight microchannel heat sink. In another experimental and numerical investigation done by Wang et al.^18^, the hydrodynamic and thermal performance of a fractal-tree-like heat exchanger has been studied. They reported that using a fractal-tree-like heat exchanger leads to improve the hydrodynamic performance, and it reduces the pressure drop. Moreover, they stated that the studied fractal-tree-like heat exchanger has a significant heat transferability. They also declared that the coefficient of performance of a fractal-tree-like heat exchanger is higher than a traditional spiral-tube heat exchanger. The heat transfer optimization of a fractal-like microchannel has been done by Wang and Mujumdar^19^. They applied a constant heat flux of 10 W/cm^2^ along the top wall of the microchannel. They compared the results of pressure drop and temperature distribution of the fractal-like microchannel with traditional straight channels and found that the temperature distribution in the fractal-like microchannel is better than the straight microchannel. They also declared the robustness of the fractal-like microchannel in the application of electronic cooling in which high reliability is needed. There are also other numerical studies on the effects of using fractal-like microchannels on temperature distribution, pressure drop, and heat transfer performance^20–25^.

In the present study, the main aims and innovations of the present study are to (1) proposing a more compact cooling system compared to the conventional cooling system, and (2) providing a more uniform temperature distribution on the surface of IGBTs and diodes, cool downing the PE module below the maximum junction temperature. The influence of the constructal theory on temperature distribution, Nu number, pressure loss, and heat transfer performance has been studied. Two leaf-inspired microchannels with different channels’ configurations have been studied over a different ranges of Re number, and the results have been compared with a straight conventional MCHS.

## Problem statement

**Figure 1:**
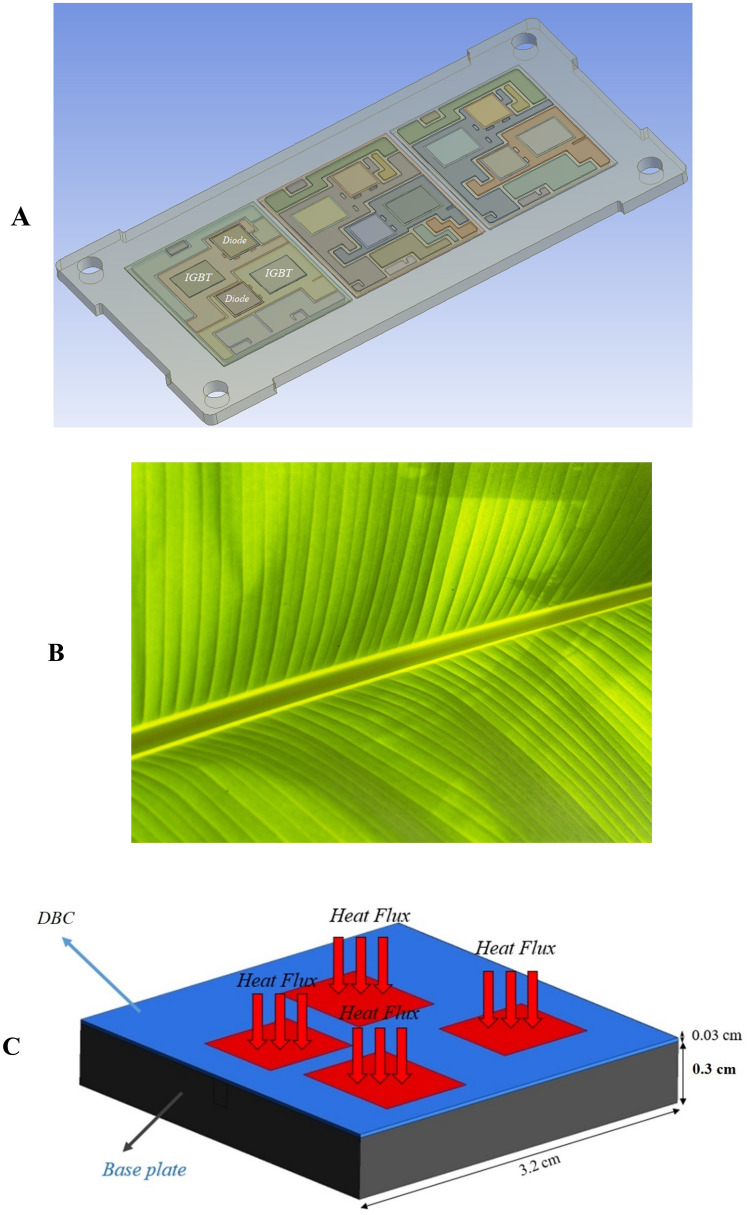
(**A**) A 3D CAD view of the studied power module (3D model created by CATIA softwar, www.3ds.com), (**B**) natural structure of a leaf (lateral vein structure), and (**C**) A schematic view of the geometry of the proposed cooling system (Created by Mircrosoft PowerPoint, www.microsoft.com).

In the present study, the main aim is to cool down a PE module (1200 V and 75 A) and provide a uniform temperature distribution on the surface of the IGBTs and diodes. Figure 1A shows a 3D view of the studied PE module, which is a commercial Si IGBT with the voltage and current of 1200 V and 75 A, respectively. The thermal stack of the studied module includes a direct bonded copper (DBC) substrate, Sn3.0Ag0.5Cu (SAC305) solder joints, and the copper base plate. The detailed information of each layer has been presented in Table 1. The heat flux generated by each IGBT and diode is assumed as 100 W/cm^2^. A microchannel, which is inspired by the excellent natural structure of a leaf (Fig. 1B), has been designed to achieve a more uniform temperature distribution on the chips. The influence of the constructal theory (having different inlets and outlets) on the heat transfer performance of the MCHS has been studied. Figure 1C presents a schematic view of the PE module and the designed cooling system. It must be noted that since all the three modules are the same, in the present study, one of them is simulated to reduce the cost and time of the simulation.

**Table 1:** Detailed information of each layer of the studied power module^26^. (Reprinted with the permission of Elsevier).

Layer material	Thickness (µm)	Density (kg/m^3^)	Thermal conductivity (W/m K)		Specific heat capacity (J/kg K)	
			Temperature (°C)	Value	Temperature (°C)	Value
Si die	120	2329	25	148	25	705
			125	98.9	125	788.3
			225	76.2	225	830.7
DBC Al_2_O_3_	380	3965	25	37	25	785.5
			125	27.2	125	942
			225	20.9	225	1076
Die solder	100	7370	All	57	All	220
DBC copper	300	8960	All	401	All	285
Base plate solder	250	7370	All	57	All	220
Base plate	3000	8960	All	401	All	385

**Figure 2:**
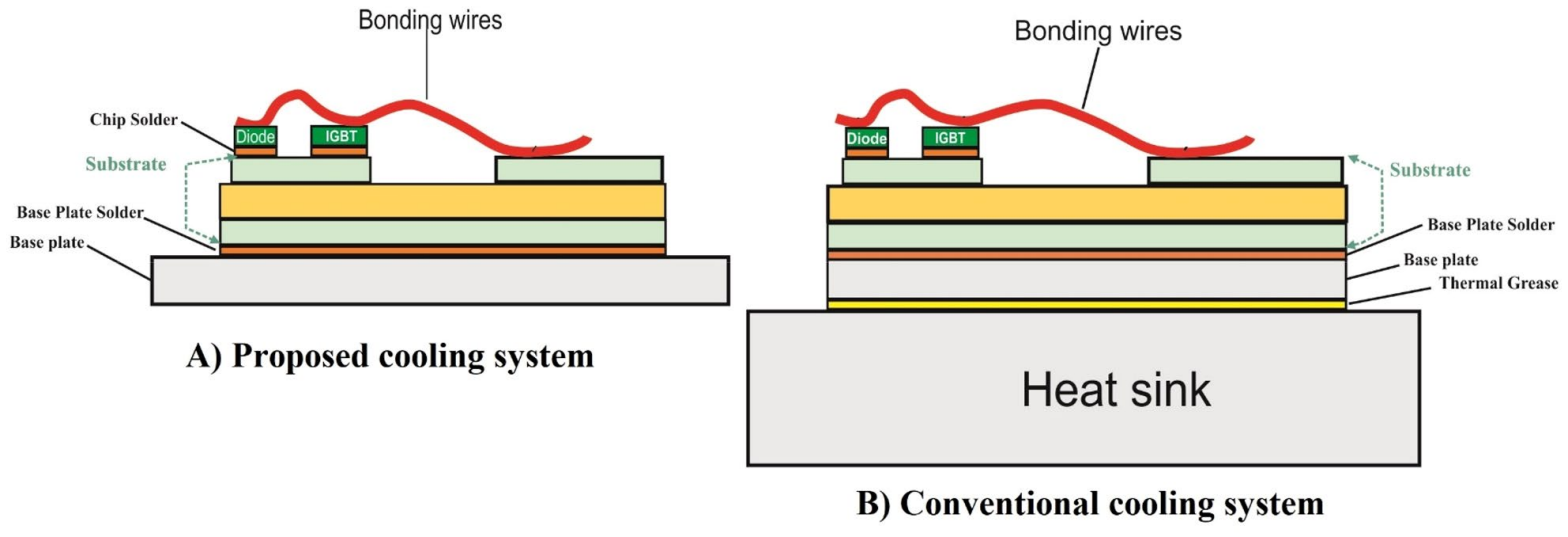
A structural comparison between (**A**) the proposed cooling system in the present study and (**B**) conventional cooling system. (Created by Mircrosoft PowerPoint, www.microsoft.com) **.**

Figure 2 depicts a view of the conventional cooling systems and the proposed cooling system in the present study. It is seen that the proposed cooling system is more compact compared to the conventional ones. Moreover, the microchannels have been designed within the base plate. This way, the thickness of the entire cooling system has been hugely reduced.

**Figure 3:**
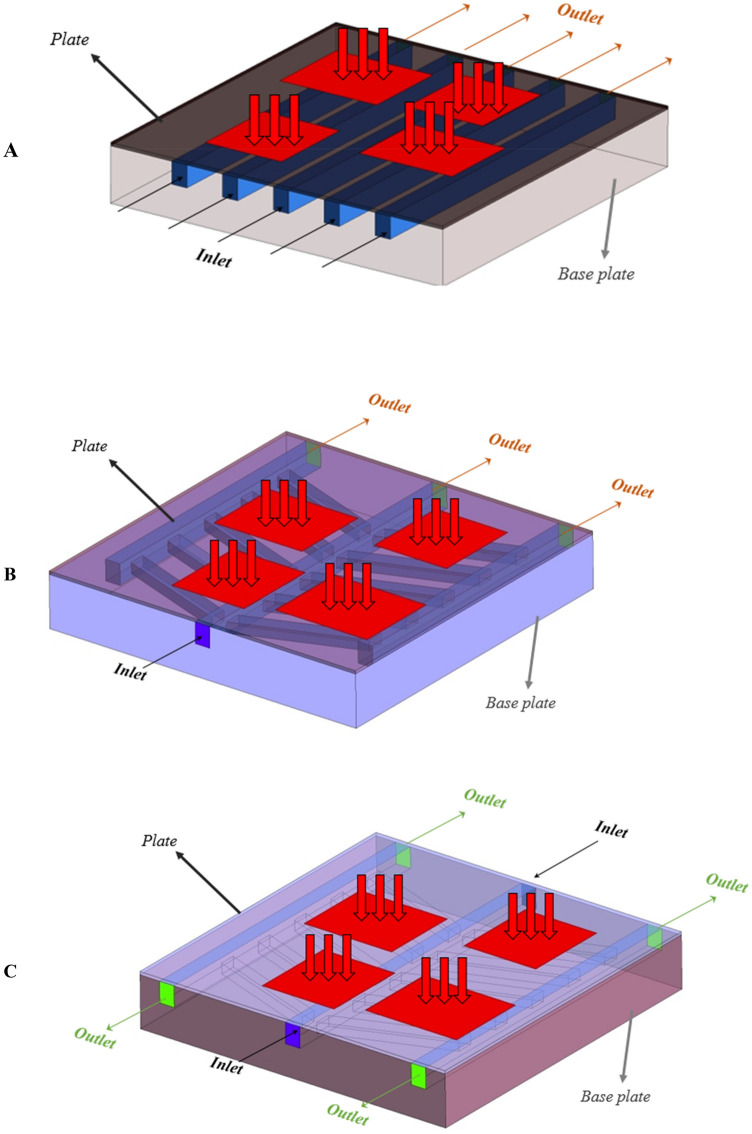
Three different studied structures of MCHS in the present study: (**A**) conventional straight MCHS, (**B**) and (**C**) leaf-inspired MCHS. (3D models created by ANSYS Workbench, www.ansys.com).

In order to show the effectiveness of the proposed leaf-inspired MCHS, the results have been compared with those of conventional straight MCHS. Thus, in the present study, three different structures have been considered. Figure 3 presents different studied structures. Figure 3A shows a conventional straight MCHS in which the fluid enters from one side of the MCHS and exits from the other side. Figure 3B shows the designed MCHS in the present study in which the fluid enters into the MCHS through one inlet located in the middle, and it exits from three different outlets on the other side of the MCHS. As for the third model, which is presented in Fig. 3C, there are two inlets on two sides of the MCHS and two outlets on each side. The flow is laminar since the studied *Re* numbers are less than 1500. It must be noted that the coolant fluid in the present study is pure water, and the volumetric flow rate is constant in all the models. Moreover, in order to investigate the effects of the channels’ arrangement on the fluid behavior and heat transfer, the wetted cross-sectional area in all the studied geometries are approximately the same.

## Computational model

### Governing equations and model assumptions

The governing equations for a 3D, steady-state, laminar, and incompressible fluid through a MCHS are continuity, momentum, and energy equations as follows:

1 $$ \frac{\partial u}{{\partial x}} + \frac{\partial v}{{\partial x}} + \frac{\partial w}{{\partial z}} = 0 $$

2 $$ \rho_{f} \left( {u\frac{\partial u}{{\partial x}} + v\frac{\partial u}{{\partial y}} + w\frac{\partial u}{{\partial z}}} \right) = - \frac{\partial P}{{\partial x}} + \mu \nabla^{2} u $$

3 $$ \rho_{f} \left( {u\frac{\partial v}{{\partial x}} + v\frac{\partial v}{{\partial y}} + w\frac{\partial v}{{\partial z}}} \right) = - \frac{\partial P}{{\partial y}} + \mu \nabla^{2} v $$

4 $$ \rho_{f} \left( {u\frac{\partial w}{{\partial x}} + v\frac{\partial w}{{\partial y}} + w\frac{\partial w}{{\partial z}}} \right) = - \frac{\partial P}{{\partial z}} + \mu \nabla^{2} w $$

The Re number can be calculated using the following equation considering the hydraulic diameter of the channel inlet and thermophysical properties of water at 25 °C:

5 $$ Re = \frac{\rho \,u\,D}{\mu } $$

The convective heat transfer coefficient can be calculated as:

6 $$ h = \frac{{q_{w} }}{{\left( {T_{w} - T_{b} } \right)}} $$

Then, the Nu number can be calculated as:

7 $$ Nu = \frac{h\,D}{k} $$

The pressure drop, which is a function of velocity and friction factor, can be calculated as:

8 $$ f = 2\Delta P\frac{D}{L}\frac{1}{{\rho \,u^{2} }}\,\,\, \to \,\Delta P = \frac{{L\rho \,u^{2} }}{2D}f $$

### Boundary conditions and numerical procedure

In the present study, the inlet mass flow rate boundary condition for the inlet fluid and the outlet pressure boundary condition for the fluid outlet is applied. Moreover, the constant heat flux boundary condition is considered for the IGBTs and diodes, and the walls have been assumed as insulated walls. Figure 3 presents the boundary conditions for each of the studied cases. To perform the numerical simulation (solving the governing equations), the ANSYS FLUENT 19.0, which is a CFD commercial code, have been employed. It must be noted that the second-order upwind method has been employed for discretizing the governing equations, and the pressure-based solver has also been used. Moreover, the Semi-Implicit Method for Pressure-Linked Equations (SIMPLE) algorithm has been employed for coupling the velocity and pressure. It is noteworthy to mention that the convergence of 10^–4^ has been considered for the residual of mass and momentum equation, and the convergence of 10^–7^ has been considered for the residual of the energy equation.

## Results and discussion

### Validation and grid independence

**Figure 4:**
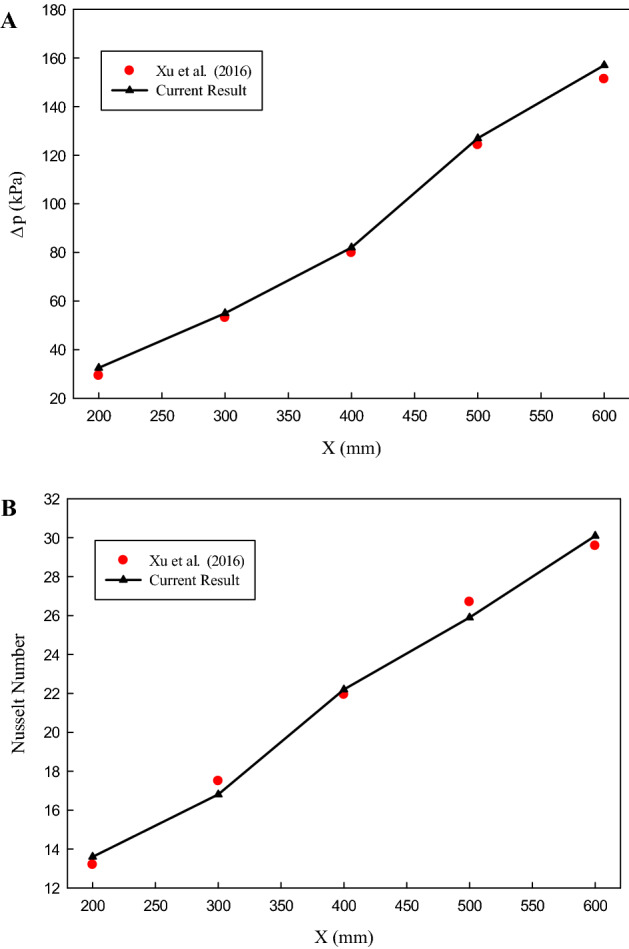
The results of the validation study.

Ensuring the accuracy of the numerical results, a comparison between the numerical results and those of the experimental results presented by Xu et al.^21^ has been made. They have studied the flow and heat transfer in a fractal silicon MCHS. Their experimental work has been simulated based on the presented boundary conditions and numerical procedure in Sect. 3. In Fig. 4 the results of comparing the *Nu* number and pressure drop achieved in the present study with the experimental results of Xu et al.^21^ have been presented. As can be seen, there is an excellent agreement between the experimental data and the numerical results, which proves the accuracy of the numerical method employed in the present study.

**Figure 5:**
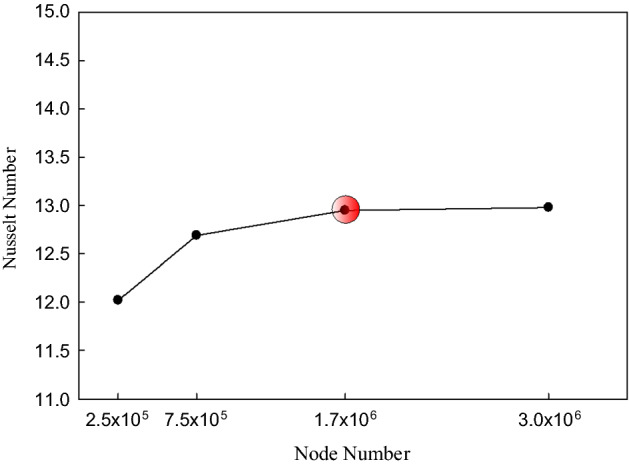
The results of the grid independence.

Since in the numerical simulation, the number of elements has certain effects on the results, it is necessary to study the grid independence and find the optimum number of elements to make sure the results are independent of the number of elements. It should be noted that the grid independence is considered for the error in *Nu* number to be below 10^–7^. To do this, the grid independence has been studied by considering the *Nu* number in the conventional straight microchannel (Fig. 3A) at the volumetric flow rate of 0.0132 kg/s, and the results are presented in Fig. 5. As can be seen, increasing the number of elements to more than 1.7 million, the *Nu* number, which is highly sensitive to the density of the elements near the walls, showed no considerable changes. Thus, in the rest of the simulations, this number of elements have been used.

### Chip surface temperature profile for the studied configurations

**Figure 6:**
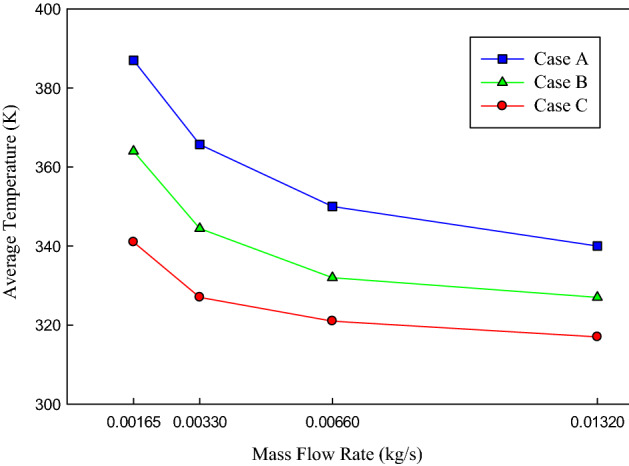
The variations of the average chips’ temperature concerning the mass flow rate of the coolant in three different channel configurations.

As it is stated before, the main aim of the present study is to uniformly cool down the IGBT and diode chips. It is of paramount importance to provide uniform temperature distribution on the chips and cooling down the chips to temperatures less than 120 °C. Thus, the surface temperature profile of the chips (IGBTs and diodes) are of paramount importance. It is expected that the channels’ configuration has certain effects on the temperature distribution on the chips. The average temperature of the chips versus the mass flow rate in three studied configurations has been presented in Fig. 6. It can be seen that increasing the mass flow rate of the coolant resulted in decreasing the average temperature of the chips. Decreasing the average temperature means that the heat transfer rate between the heated surface and the coolant inside the channels is enhanced. This is because of the fact that increasing the mass flow rate of the coolant leads to increasing the heat capacity to absorb the heat from the chips. It is also inferred from Fig. 6 that in a constant mass flow rate of the coolant, the minimum and maximum chip temperature took place in case C and A, respectively. Thus, it can be concluded that the configuration of the case C is more suitable to reduce the chips’ temperature. That is, in the case with the minimum mass flow rate of 0.00165 (kg/s), the chips’ temperature in cases A, B, and C is 387, 364, and 341 K, respectively. In other words, by changing the channels’ configuration from A to C, and with a constant mass flow rate, the average chips’ temperature decreased by 46 K. Moreover, the effect of changing the channels’ configuration from A to C on the chips’ temperature at the maximum mass flow rate of 0.0132 (kg/s) is a decrease of 23 K in the chips’ temperature.

**Figure 7:**
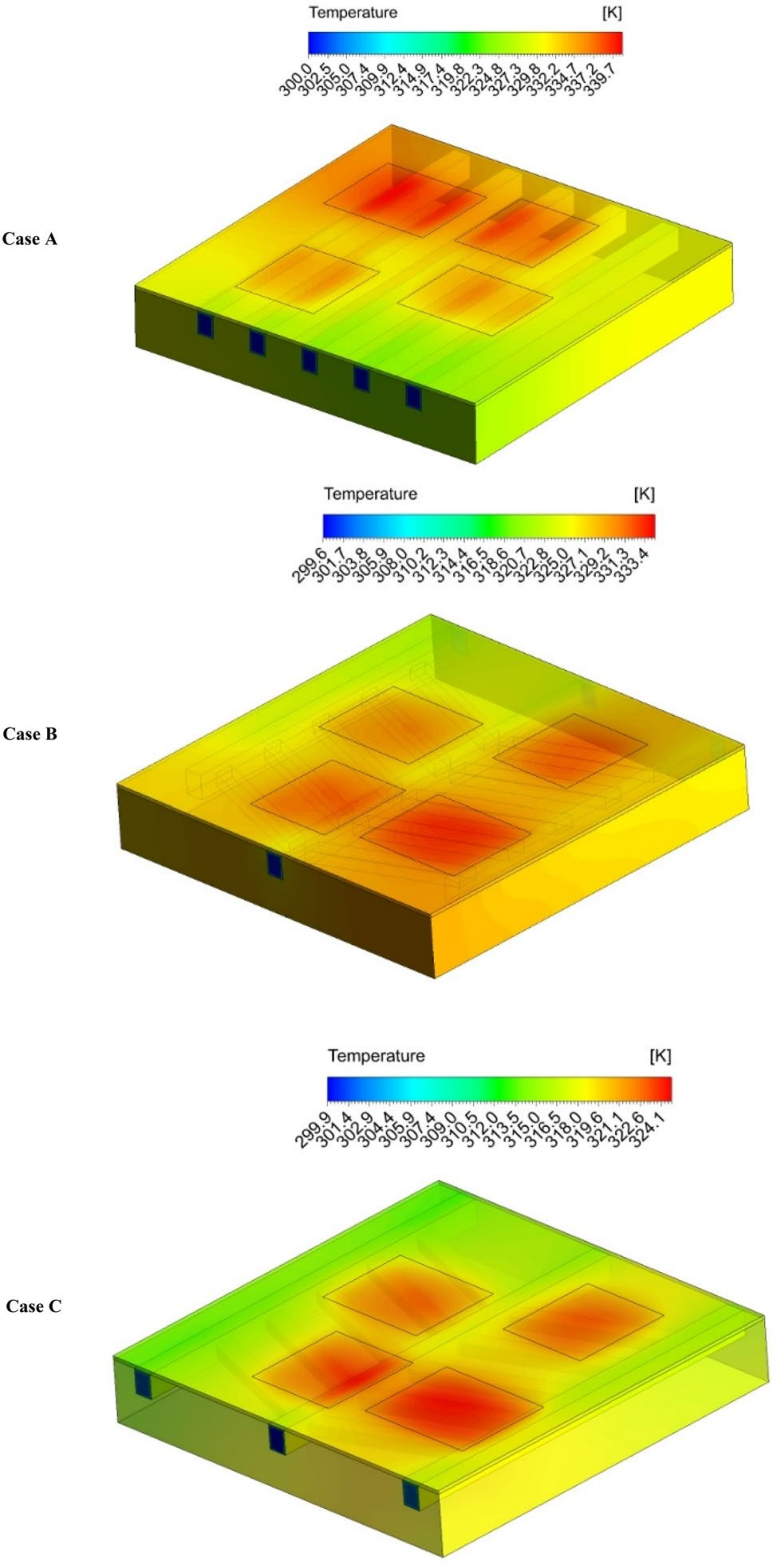
The contours of temperature for the all the considered configurations in the highest considered mass flow rate (0.0132 kg/s). (3D models created by ANSYS Workbench, www.ansys.com).

Figure 7 depicts the temperature distribution in the three studied channel configurations in the maximum mass flow rate of 0.0132 (kg/s). As can be seen, in the conventional straight channel configuration (case A), which has five inlets and five outlets, the two chips located close to the inlets have lower temperatures compared to the chips close to the outlets. As for the case B, which is a leaf-inspired configuration with one inlet and three outlets, the maximum chip temperature is decreased compared to the case A. Moreover, the cooling process is more effective compared to case A, and the chips close to the outlets received a better cooling. Interestingly, the temperature contour of case C shows that in this configuration that has one inlet from each side (two inlets in total) and two outlets in each side (four outlets in total), the maximum temperature dropped considerably, and the temperature distribution on the chips is better than other configurations. This can be better understood by presenting the effects of different configurations on the streamlines distribution, which will be presented in the following sections.

**Figure 8:**
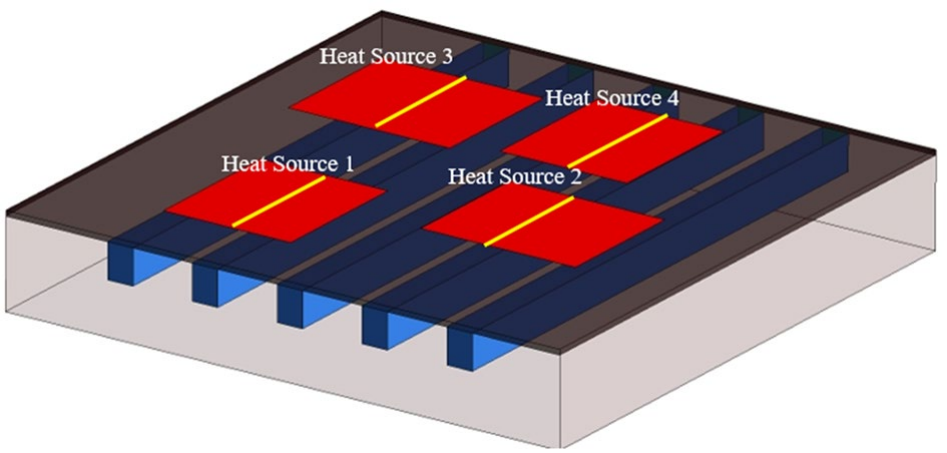
The location of the centerlines on the surface of the chips. (3D models created by ANSYS Workbench, www.ansys.com).

Providing a better understanding of the effects of different studied channels’ configurations on the temperature distribution, the temperature distribution on the chips’ surface has been scrutinized at the Highest mass flow rate of 0.0132 kg/s. Figure 8 illustrats the centerlines on the surface of the chips.

**Figure 9:**
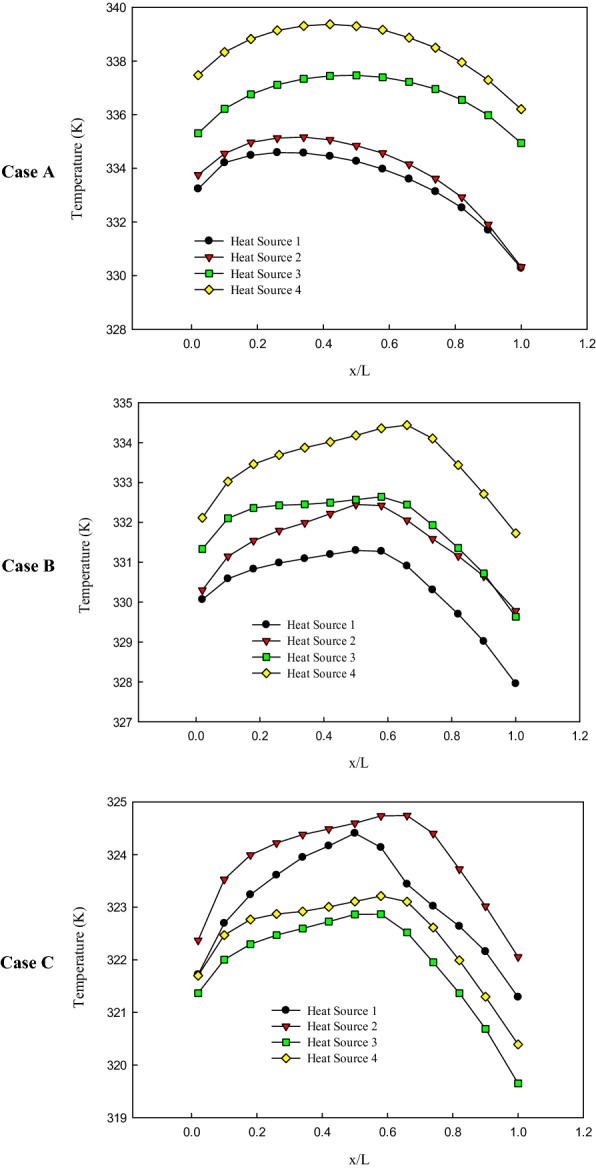
The distribution of temperature on the centerline of the chips at the highest considered mass flow rate (0.0132 kg/s).

The variations of the temperature distribution on the surface of the chips, for all the studied channels’ configurations, have been presented in Fig. 9. As can be seen, in case A, the minimum and maximum temperatures are in chip 1 and 4, respectively. It shows that the temperature of the coolant is increased along the channels (from inlet to outlet), which leads to decreasing the cooling performance of the system. Moreover, it can be seen that the maximum temperature of chip 4, which is located close to the outlets, is 5 K higher than chip 1, which is located close to the inlets.

As for case B, which is a leaf-inspired configuration with one inlet and three outlets, the temperature distribution on the chips showed that the chip 1 and 2, which is located close to the inlet, possess lower temperature compared to the chips located close to the outlets (chip 3 and 4). It should be noted that the temperature of the chips has been decreased in this case compared to case A.

The temperature distribution on the chips’ surface in case C, which is a leaf-inspired configuration with two inlets and four outlets, showed that the maximum temperature on the hottest chip has been considerably decreased compared to the other configurations (case A and B); the hottest chip is 15 K cooler in this case compared to other cases. It is interesting to note that unlike the case A and B, in case C, chips 3 and 4 possess lower temperature compared to chip 1 and 2.

### Nusselt number

**Figure 10:**
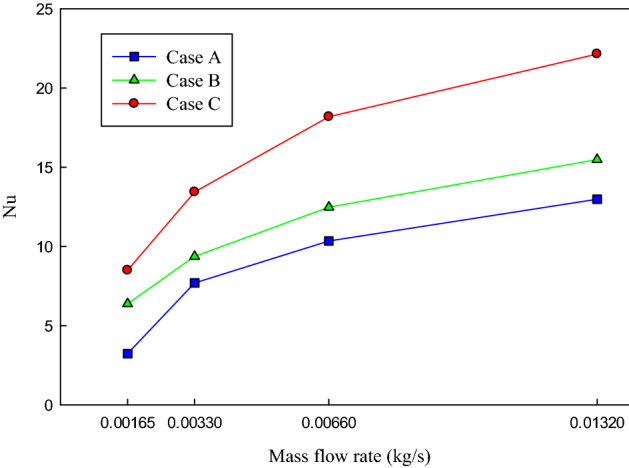
The variations of the Nu number concerning the mass flow rate of the coolant in the three studied channel configurations.

In the following, the results of the effects of flow rate and the structure of the channels on heat transfer performance of the MCHS will be presented and discussed. Figure 10 presents the effects of the mass flow rate variations and the channels’ configuration on the *Nu* number. According to the presented results of the average chips’ temperature, it is expected that the case C that possesses the minimum working temperature, shows the maximum *Nu* number. As can be seen in Fig. 10, the maximum *Nu* number is achieved in case C. It can also be seen that increasing the mass flow rate resulted in increasing the *Nu* number in all the studied cases.

### Pressure loss

Since changing the channels’ configurations from the straight conventional channels (case A) to leaf-inspired configurations (case B and C) will be accompanied with increasing the pressure loss, it is necessary to investigate the effects of changing the channels’ configurations on the pressure loss in all the studied mass flow rates. In the following, the effects of changing the channels’ configuration on the pressure loss will be investigated and discussed.

**Figure 11:**
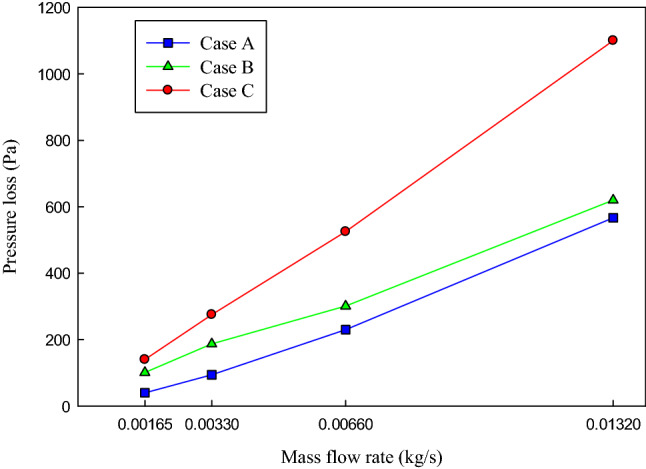
The variations of the pressure loss concerning the mass flow rate of the coolant in the three studied channel configurations.

The variations of the pressure loss concerning the mass flow rate in three studied channels’ configurations have been presented in Fig. 11. As can be seen, increasing the mass flow rate resulted in increasing the pressure loss in all the studied configurations. The reason is that increasing the mass flow rate means increasing the velocity of the fluid, which leads to increasing the pumping power. Investigating the effects of changing the channels’ configurations in a constant mass flow rate showed that the pressure loss increases along the channels. That is, in a constant mass flow rate of 0.00165 (kg/s), the pressure loss in configuration A is 40 Pa while it is 140 Pa in configuration C (leaf-inspired configuration). To put it in another way, changing the channels’ configuration from A to C results in increasing the pressure loss by 3.5 times. Moreover, the pressure loss increased by 1.9 times in the maximum studied mass flow rate by changing the configuration from A to C.

**Figure 12:**
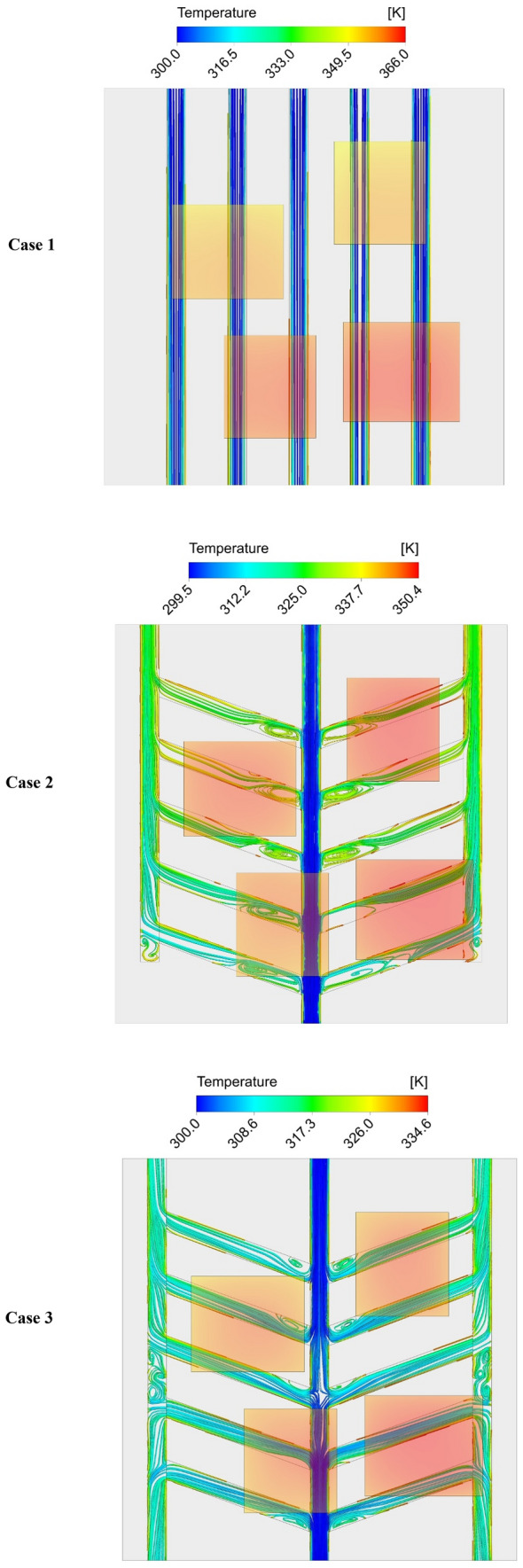
The variations of streamlines in the three studied channels configurations at the mass flow rate of 0.003 kg/s. (created by ANSYS Workbench, www.ansys.com).

Providing a better understanding to readers on the effects of the channels’ configurations on the fluid flow, it would be helpful to investigate the flow streamlines. Figure 12 presents the flow streamlines in a constant mass flow rate of 0.0132 (kg/s) of all the three studied configurations. As can be seen, in case A, which is a conventional straight MCHS with five inlets and five outlets, the flow streamlines are parallel throughout the inlet and outlets. As for case B in which there are one inlet and three outlets, it can be seen that there are some vortexes in the channels that leads to an undesirable pressure gradient. This is the main reason of increasing the pressure loss in case B compared to case A. In case C in which the fluid enters to the MCHS from one inlet located in each side (two inlets in total), and there are two outlets on each side (four outlets in total), investigating the streamlines show that with the collision of two flows in the center of the channel, the flow has been distributed in the interface channels. As a result, in addition to the vortexes in the interface channels like the case B, since the flow is divided into two parts to exit the MCHS, more vortexes in the middle of the side channels are created. Thus, based on the flow streamlines, it can be concluded that the maximum pressure loss will be in case C, which approves the results presented in Fig. 11.

In the present study, it is of paramount importance to study the temperature distribution on the chip and as well as providing a unform temperature distribution on the chips’ surface. Thus, the term of “*standard temperature deviation* (*σ*_*T*_)”, which would be a perfect criterion study the distribution of temperature on the surface of the chips, is defined as follows:

9 $$ \begin{aligned} \overline{T} & = \frac{1}{n}\sum\limits_{i = 1}^{n} {T_{i} } \\\upsigma _{T} & = \sqrt {\frac{1}{n}\sum\limits_{i = 1}^{n} {(T_{i} - \overline{T} )^{2} } } \\ \end{aligned} $$

where *T*_*i*_, $\overline{T}$, and *n* represent the temperature of each point located on the centerline of the chips, the average temperature on the centerline of the chips, and the number of nodes on the centerline of the chips, respectively.

**Figure 13:**
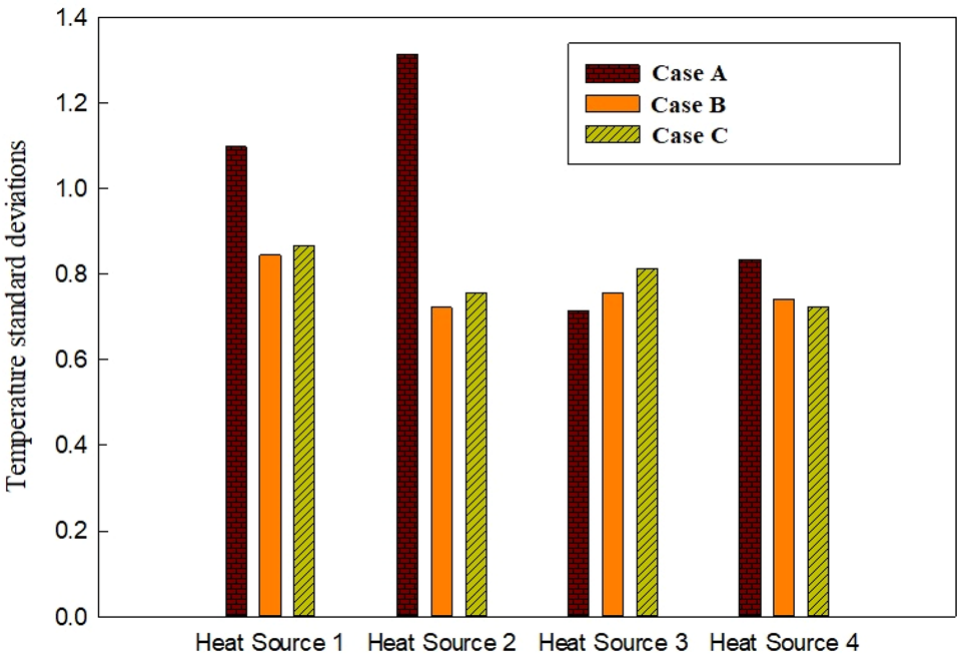
The variations of *σ*_*T*_ on the chips’ surface in all the considered configurations at the highest mass flow rate (0.0132 kg/s).

The variations of the *σ*_*T*_ on the chips’ surface in all the considered cases at the highest mass flow rate have been presented in Fig. 13. It is revealed before that the heat sources 1 and 2 are working in lower temperatures compared to heat sources 3 and 4 since they are located close to the inlets in the case A. However, based on Fig. 3, it can be seen that the temperature distribution on the surface of the heat sources 1 and 2 in case A is lower than the two other cases. Thus, it can be concluded that case A is not suitable for having a uniform temperature distribution on the surface of the chips. Moreover, the average temperature in case A is also higher than the other cases.

**Figure 14:**
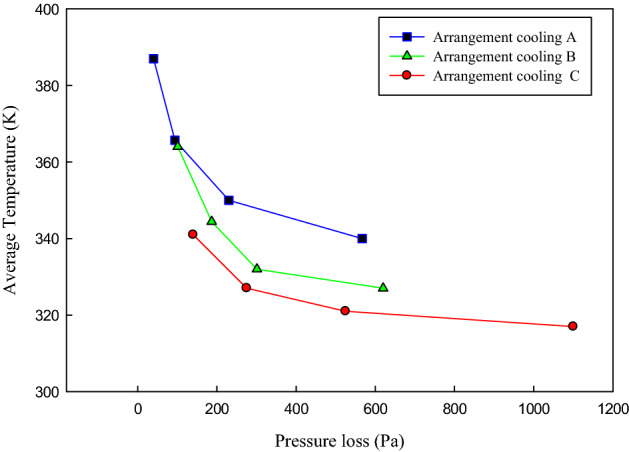
The variations of the average temperature of the chips concerning pressure loss in the three studied channel configurations.

The performance of the studied cooling systems on the flow uniformity has also been investigated, and it is found that the performance of cases B and C is approximately similar. Figure 14 shows the variations of the average chips’ temperature concerning pressure loss in all the three studied configurations. Based on this figure, it can be concluded that case B has a better performance in decreasing the chips’ temperature with the lower pressure loss compared to case C. Thus case B is recommended in the present study in which by having the minimum pressure loss compared to the case A (the pressure drop of 53 Pa higher than case A), the average chips’ temperature is decreased by 14 K.

## Concluding remarks

The thermal management of a PE system has been studied in the present study. The main objective was to provide a uniform temperature distribution on the IGBT and diode chips’ surface. To do that, a new MCHS, which is inspired by the unique natural structure of a leaf, has been designed, and the fluid flow and heat transfer have been simulated employing the commercial CFD code of ANSYS FLUENT 19.0. Two different channels configurations (case B and C) have been studied, and the results have been compared with a straight conventional MCHS (case A) under the same boundary conditions. The effects of channels’ configuration on the temperature distribution on the chips’ surface, *Nu* number, and pressure loss have been investigated. The results revealed that in case A, the temperature of the chips located close to the inlets are lower than those chips located close to the outlets. Moreover, the temperature is not uniformly distributed on the surface of the chips. As for cases B and C, the performance of these leaf-inspired configurations showed that the average chips’ temperature in case C is lower than case B by 10 K. However, the pressure loss in case C is approximately two times greater than case B. Thus, based on the heat transfer performance and the pressure loss, it can be concluded that for cooling down the studied PE system, the leaf-inspired configuration of case B that comprises of one inlet and three outlets is the best configuration among other two configurations (case A and C).

It would be a good idea to investigate the effects of having secondary channels and employing nanofluid, which has superior thermal properties compared to water, as the coolant for future works. Moreover, the geometry optimization would be performed to achieve the optimized geometry, which results in having more uniform temperature distribution on the chips surface and minimum pressure loss.
